# The influence of operation time for hip hemiarthroplasty on complication rates and mortality in patients with femoral neck fracture: a retrospective data analysis

**DOI:** 10.1186/s13018-024-04797-7

**Published:** 2024-05-27

**Authors:** Nikolai Ramadanov, Mikhail Salzmann, Maximilian Voss, Robert Hable, Hassan Tarek Hakam, Robert Prill, Dobromir Dimitrov, Roland Becker

**Affiliations:** 1 Center of Orthopaedics and Traumatology, University Hospital Brandenburg/Havel, Brandenburg Medical School Theodor Fontane, 14770 Brandenburg an der Havel, Germany; 2 Faculty of Health Science Brandenburg, Brandenburg Medical School Theodor Fontane, Brandenburg an der Havel, Germany; 3 Faculty of Applied Computer Science, Deggendorf Institute of Technology, Deggendorf, Germany; 4 Department of Surgical Propedeutics, Faculty of Medicine, Medical University of Pleven, Pleven, Bulgaria

## Abstract

**Background:**

The aim of the present study was to investigate the influence of various factors, in particular operation time, on mortality and complication rates in patients with femoral neck fractures who have undergone hip hemiarthroplasty (HHA) and to determine a cut-off value above which mortality and complication rates increase significantly.

**Methods:**

Cases of patients with femoral neck fracture treated with HHA between 1 January 2017 and 31 December 2023 were screened for eligibility. Multiple logistic regressions were calculated to determine which factors (patient age, experience of surgeon, patient sex, ASA score, time to surgery, operation time) influenced the incidence of complications and mortality. The exact cut-off value for complications and mortality was determined using the Youden index of the ROC curve (sensitivity vs. specificity) of logistic regression.

**Results:**

A total of 552 patients were considered eligible for this study. During the 90-day follow-up period after HHA, 50 deaths and 34 complications were recorded, giving a mortality rate of 9.1%, and a complication rate of 6.2%. Of the 34 complications recorded, 32.3% were infections, 14.7% dislocations, 20.7% trochanteric avulsions, 11.8% periprosthetic fractures, 11.8% nerve injuries, and 8.8% deep vein thrombosis. The odds ratio (OR) of a patient experiencing a complication is 2.2% higher for every minute increase in operation time (Exponential Beta − 1 = 0.022; *p* = 0.0363). The OR of a patient dying is 8.8% higher for each year increase in age (Exponential Beta − 1 = 0.088; *p* = 0.0007). When surgery was performed by a certified orthopaedic surgeon the mortality rate lowered by 61.5% in comparison to the surgery performed by a trainee (1 – Exponential Beta = 0.594; *p* = 0.0120). Male patients have a 168.7% higher OR for mortality than female patients (Exponential Beta − 1 = 1.687; *p* = 0.0017). Patients with an operation time of ≥ 86 min. have a 111.8% higher OR for mortality than patients with an operation time of < 86 min. (Exponential Beta – 1 = 1.118).

**Conclusion:**

This retrospective data analysis found that the risk of a patient experiencing a complication was 2.2% higher for every minute increase in operation time. Patients with an operation time above the cut-off of 86 min had a 111.8% higher risk of mortality than those with an operation time below the cut-off. Other influencing factors that operators should be aware of include patient age, male sex, and operator experience.

**Supplementary Information:**

The online version contains supplementary material available at 10.1186/s13018-024-04797-7.

## Introduction

Hip fractures are very common in older people [1, 2]. About 90% of hip fractures occur in people over the age of 65 [1, 2]. Patient mortality after a hip fracture ranges from 10% in the first month to 30% in the first year [1, 2]. Femoral neck fractures in elderly patients are often treated with either total hip arthroplasty (THA) or hip hemiarthroplasty (HHA), as this type of surgical treatment has lower complication rates and better functional outcomes compared with head preservation surgery [3]. Depending on the patient’s activity level and co-morbidities the surgeons choose between HHA and THA [3, 4]. Due to the aging of the average population in developed countries, the number of femoral neck fractures is predicted to increase in the coming decades. Complications following femoral neck fractures often have a devastating effect on the already elderly population, often suffering from severe comorbidities. There is a high mortality rate of 20.5%, within the first year after a femoral neck fracture [5]. Based on this outcome there is a strong interest in reducing the rate of complications and mortality as much as possible. In addition to the correct positioning of the prosthesis components, the surgeon’s contribution is to maintain an adequate operation time. The current state of scientific knowledge is mainly limited to the investigation of correlations between mortality/complications and operation time in elective THA. The scientific community predominantly agrees that longer operation time performing THA is associated with higher complication rates [6–11]. It has been reported in an analysis of 103,044 patients undergoing THA, that exceeding operation times of 90 min. were associated with higher infection rates [7]. Another analysis of 99,444 patients undergoing total joint arthroplasty showed that an operation time lasting more than two hours was associated with higher short-term morbidity and mortality [9]. Furthermore, an analysis of 65,474 patients undergoing total joint arthroplasty found that each 15-minute increase in operation time increased the risk of blood transfusion, wound dehiscence, renal failure, sepsis, wound infection, urinary tract infection, and hospital readmission [10]. A low-risk operation time of approximately 80 min. for THA, has been suggested in an analysis of 89,802 cases [11]. In contrast to this robust evidence, mortality and complications in patients with femoral neck fractures treated with HHA appear to have been understudied.

The aim of the present study was to investigate the influence of various factors and, above all, the operation time on the mortality and complication rates in patients with femoral neck fractures operated on with HHA. Furthermore, an attempt was to be made to determine a cut-off value above which the mortality and complication rates increase significantly. It was hypothesized that the complication and mortality rates would increase after prolonged operation time.

## Methods

### Data collection and processing

This retrospective study was prepared according to checklists and reporting guidelines for research in orthopaedics [12]. The institutional registry of the University Hospital of Brandenburg/Havel was screened for cases of patients with femoral neck fracture treated with HHA between 1 January 2017 and 31 December 2023. Ethical approval was given by the Ethical Committee of the University of Brandenburg (No. 79,022,024-BO-E-RETRO). Patients with multiple trauma, pathological fractures, and patients with femoral neck fractures treated with THA were excluded. Data extraction was performed independently by two researchers (NR and MV) from the hospital information system. All operative reports, anesthesia records, discharge letters and quality assurance data sheets were reviewed. The data extraction sheets were then compared and inaccuracies were resolved by consensus between the two researchers (NR and MV). The following data were extracted: patient date of birth and sex, patient height, and weight, patient ASA score, operator experience, time from admission to surgery, operation time, patient death, and complications. Patient age and body mass index (BMI) were calculated from the extracted data. The extracted data on operation time were averaged into 10 min. intervals, starting from 30 to 39 min. Patients were allocated in two groups, stratified by age. The first patient age group were patients younger than 81 years and the second group were patients older than 80 years. The time from hospital admission to surgery was defined as the time to surgery. The extracted data concerning time to surgery were divided into two groups: one group with time to surgery less than 24 h after hospital admission and another group with time to surgery later than 24 h after hospital admission.

### Complications and mortality

Data of the following complications were analyzed in the study: infection, dislocation, trochanteric avulsion, periprosthetic fracture, nerve injury, deep vein thrombosis. In addition, all deaths of patients who had undergone surgery, regardless of the specific cause of death were recorded. The observation period for complications and mortality in the patients included in this retrospective study was 90 days after surgery in each case.

### Statistics

Categorical data are presented as absolute and relative frequencies, continuous data as mean and standard deviation. Complications and mortality were the two primary endpoints and therefore binary outcomes. The patient cohort was presented in detail in a table based on descriptive statistics. Multiple logistic regressions were calculated to determine which factors (patient age, patient age group, experience of surgeon, patient sex, ASA score, time to surgery, operation time, rounded operation time) influenced the incidence of complications and mortality. To determine a cut-off value with an increase in both endpoints, the frequencies of complications and mortality were first calculated for the rounded operation times and presented in corresponding graphs. The exact cut-off value for complications and mortality was determined using the Youden index of the ROC curve (sensitivity vs. specificity) of logistic regression. Once the cut-off values for mortality and complications had been determined, they were checked again using logistic regression to see whether patients with an operation time above the cut-off value actually had a significantly higher risk of mortality or complications. The statistical calculations were carried out by a professional statistician (RH) using R version 4.2.1.

## Results

### Patient selection

A total of 755 patients with femoral neck fractures were treated between 2017 and 2023. Of these patients, 203 patients were excluded for the following reasons: 182 patients underwent THA, one patient had multiple trauma, two patients had a pathological fracture, and 18 patients were treated conservatively. A total of 552 patients with femoral neck fractures underwent HHA surgery and were considered eligible for this study.

### Patient cohort

Of the 552 patients included, 525 (95.1%) underwent cemented HHA and 27 (4.9%) uncemented HHA. The mean age of the patient cohort was 84 *±* 7.6 years (range: 50–100 years). In the patient cohort, 72.5% were older than 80 years, and 33.5% were male. The mean BMI was 24.9 *±* 4.9 kg/m² (range: 14.1–50.8 kg/m^2^). According to the ASA classification, the patient cohort had the following ASA scores: ASA 1: 0.2%; ASA 2: 11.9%; ASA 3: 77.1%; ASA 4: 10.6%; ASA 5: 0.2%. Surgery was performed within 24 h of hospital admission in 66.7% of cases. Patients were operated on by a certified orthopaedic surgeon in 81.3% of cases. The remaining cases were operated by a trainee and assisted by a certified orthopaedic surgeon. The mean operation time was 68.1 *±* 16 min. (range: 37–146 min.).

### Mortality and complications

We recorded 50 deaths and 34 complications in 552 patients during the 90-day follow-up period after HHA, giving a mortality rate of 9.1% and a complication rate of 6.2%. Of the 50 deaths recorded, five patients (10%) required resuscitation during or immediately after surgery, with bone cement implantation syndrome (BCIS) suspected as the cause of subsequent death. Of the 34 complications recorded, 32.3% were infections, 14.7% dislocations, 20.7% trochanteric avulsions, 11.8% periprosthetic fractures, 11.8% nerve injuries, and 8.8% deep vein thrombosis (Table 1).

**Table 1:** Frequency of the complications and mortality

Analyzed endpoints		
	*N*	*% of 552 patients*
Mortality	50	9.1
Complication overall	34	6.2
**Types of complications**		
	***N***	***% of 34 patients***
Infection	11	32.3
Dislocation	5	14.7
Trochanter avulsion	7	20.7
Periprosthetic fracture	4	11.8
Nerv injury	4	11.8
DVT	3	8.8

### Factors influencing the mortality and complication rate

The influence of patient age, patient age group, surgeon experience, patient sex, ASA score, time to surgery, operation time, and rounded operation time on mortality and complication rates was calculated using multiple logistic regressions. As the factors ‘patient age’ and ‘patient age group’ correspond to each other, it was not possible to include both factors in the same model. The same applies to the factors ‘operation time’ and ‘operation time rounded’. Therefore, four alternative models were used for calculation of potential combinations of factors. A representative model for complication and mortality is shown in Table 2. All other models are available in the Supplementary Appendix. Initial calculations showed that BMI had no influence on complication rates and mortality. However, BMI had a high number of missing values (about 19%), which was problematic for further calculations. To ensure reliable and consistent results, BMI was excluded from further analyses. Both, operation time and rounded operation time, showed a significant influence on the complication rate in every multiple logistic regression model (Table 2 and Supplementary Appendix). The odds ratio (OR) of a patient experiencing a complication is 2.2% higher for every minute increase in operation time (Exponential Beta − 1 = 0.022; *p* = 0.0363). Patient age, patient age group, surgeon experience, patient sex, ASA score, and time to surgery had no significant influence on complication rates (Table 2 and Supplementary Appendix). Patient age, surgeon experience, and patient sex showed a significant influence on the mortality rate in every multiple logistic regression model (Table 2 and Supplementary Appendix). The OR of a patient dying is 8.8% higher for each year increase in age (Exponential Beta − 1 = 0.088; *p* = 0.0007). When surgery was performed by a certified orthopaedic surgeon the mortality rate lowered by 61.5% in comparison to the surgery performed by a trainee (1 – Exponential Beta = 0.594; *p* = 0.0120). Male patients have a 168.7% higher OR for mortality than female patients (Exponential Beta − 1 = 1.687; *p* = 0.0017). ASA score, time to surgery, and operation time had no significant influence on mortality rates (Table 2 and Supplementary Appendix).

**Table 2:** Multiple logistic regression model for complications and mortality. *CI: confidence interval*

Complication						
*Parameter*	*Exponential beta*	*95% CIs*	*Beta*	*Standard error*	*z-value*	*p-value*
Intercept	0.001	0.000-0.093	-7.257	2.489	-2.916	0.0035 **
Patient age (years)	1.009	0.966–1.053	0.009	0.022	0.397	0.6916
Senior operator: yes	1.139	0.478–2.715	0.130	0.443	0.293	0.7693
Sex: male	1.661	0.846–3.264	0.508	0.345	1.473	0.1406
ASA score	1.935	0.988–3.790	0.660	0.343	1.926	0.0541
Operation within 24 h: yes	1.202	0.588–2.458	0.184	0.365	0.505	0.6136
Operation time (min.)	1.022	1.001–1.042	0.021	0.010	2.094	0.0363 *
**Mortality**						
***Parameter***	***Exponential beta***	***95% CIs***	***Beta***	***Standard error***	***z-value***	***p-value***
Intercept	0.000	0.000-0.003	-10.785	2.570	-4.197	< 0.0001 ***
Patient age (years)	1.088	1.036–1.142	0.084	0.025	3.386	0.0007 ***
Senior operator: yes	0.406	0.201–0.820	-0.901	0.359	-2.512	0.0120 *
Sex: male	2.687	1.447–4.987	0.988	0.316	3.132	0.0017 **
ASA score	1.684	0.900-3.151	0.521	0.320	1.632	0.1027
Operation within 24 h: yes	0.805	0.430–1.505	-0.217	0.319	-0.680	0.4968
Operation time (min.)	1.002	0.981–1.022	0.002	0.010	0.155	0.8769

### Operation time

**Fig. 1:**
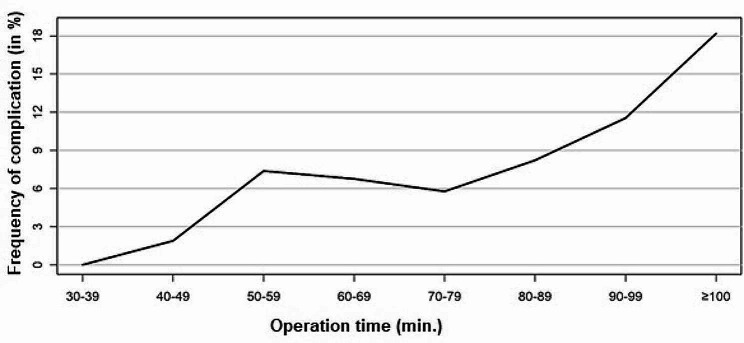
Incidence of complication rate for each 10-minute increase in operation time

**Fig. 2:**
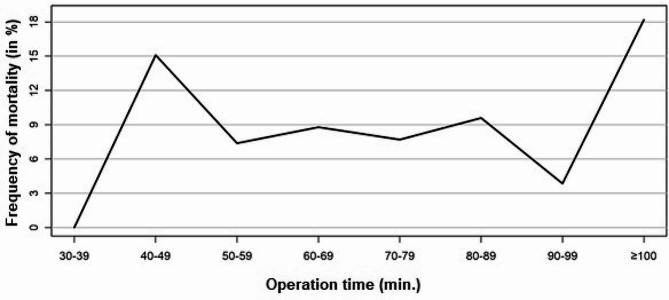
Incidence of mortality rate for each 10-minute increase in operation time

**Fig. 3:**
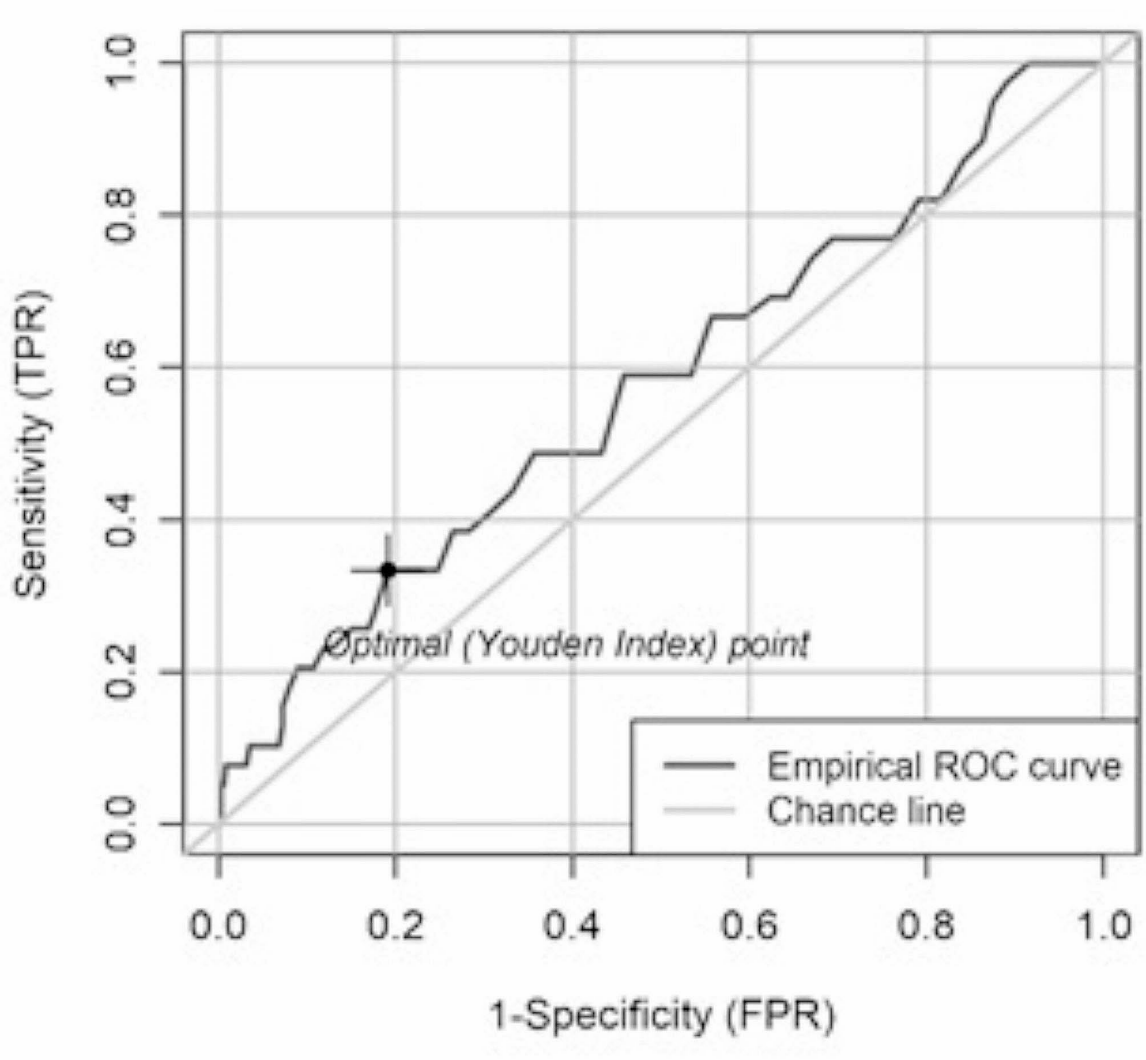
Youden index of the ROC curve with a cut-off value of 81 min. for complication

**Fig. 4:**
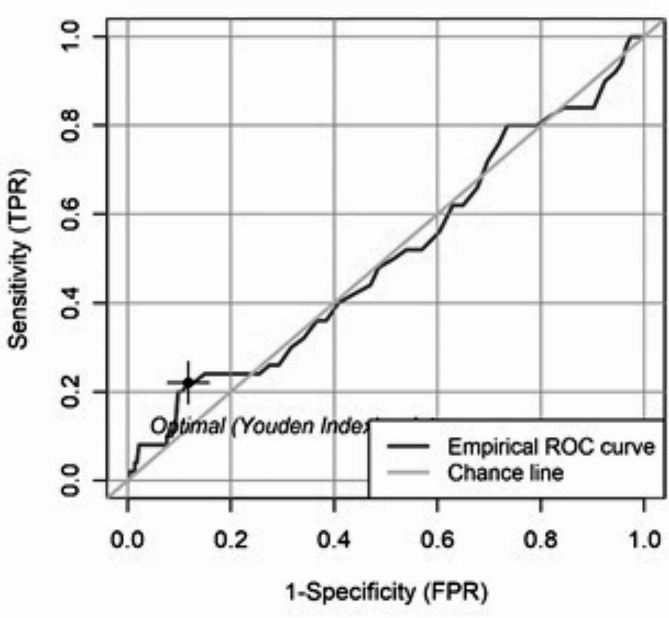
Youden index of the ROC curve with a cut-off value of 86 min. for mortality

The incidence of complications and mortality rate for each 10-minute increase in operation time is shown in Figs. 1 and 2. The cut-off value for both endpoints was determined using the Youden index of the ROC curve (sensitivity vs. specificity) of a logistic regression. The Youden index results in a cut-off value for complication at an operation time of 81 min. (Fig. 3). However, logistic regression did not show that patients with an operation time of ≥ 81 min. had a statistically significant higher risk of complications (*p* = 0.1089). The Youden index results in a cut-off value for mortality at an operation time of 86 min. (Fig. 4). Logistic regression confirmed that patients with an operation time of ≥ 86 min. had a statistically significant higher risk of mortality (*p* = 0.0417). Patients with an operation time of ≥ 86 min. have a 111.8% higher OR for mortality than patients with an operation time of < 86 min. (Exponential Beta – 1 = 1.118).

## Discussion

The main finding of this retrospective study was that the operation time shows a significant impact on the complication and mortality rate in patients who received HHA because of femoral neck fractures. Therefore the primary hypothesis can be accepted. The OR of a patient experiencing a complication is 2.2% significantly higher for every minute increase in operation time. The OR of a patient dying is 8.8% significantly higher for each year increase in age. When the operation was performed by a certified orthopaedic surgeon, the mortality rate lowered by 61.5%. Male patients showed a significantly higher mortality rate than female patients. The surgical cut-off time for increase in complications was 81 min. and the cut-off for increased mortality rate was 86 min. Patients with an operation time of ≥ 81 min. did not have a statistically significant higher risk of complications. Patients with an operation time of ≥ 86 min. had a 111.8% significantly higher OR for mortality than patients with an operation time of < 86 min.

There is a lack of comparable studies in the literature on the influence of operation time on HHA in patients with femoral neck fractures. On the other hand, a lot of literature covers the treatment of THA patient. In a National Surgical Quality Improvement Program (NSQIP) data analysis of 103,044 patients who underwent THA between 2006 and 2015, Wills et al. found a 90-minute cut-off for higher infection rates [6]. They concluded that for every 10-minute increase in operation time, the risk of infection increased [6]. Duchman et al. reported in an analysis of NSQIP data between 2011 and 2013 that operation times greater than 120 min. were associated with an increased risk of infection rates after THA and TKA [8]. Bohl et al. in an analysis of NSQIP data between 2006 and 2013, concluded that each 15-min. increase in operation time was associated with an increased risk of blood transfusion, wound dehiscence, renal failure, sepsis, wound infection, urinary tract infection, and hospital readmission [9]. In an analysis of NSQIP data from 2011 to 2015 on 89,802 patients, Surace et al. concluded that there was a strong correlation between increased operation time and perioperative complications [10]. They suggested an optimal time of around 80 min., which may be associated with a lower risk of complications following THA [10]. In an analysis of NSQIP data from 2006 to 2016 on 131,361 patients, Nowak and Schemitsch concluded that an operation time of more than 90 min. is a predictor of complications after THA and that an operation time of between 40 and 90 min. may be ideal [11].

It is important for orthopaedic surgeons to be familiar with the factors that can affect the outcome of their patients with femoral neck fractures so that they can identify patients at risk and try to manage them proactively. Geriatric traumatology as an interdisciplinary management of elderly fracture patients gains importance nowadays [13]. These patients suffer from serious medical problems and falls with subsequent femoral neck fractures are often the result. Optimization in patients care, especially prior to surgery may improve surgical outcome [14]. This is very demanding for some fractures, considering the guideline for the treatment of femoral neck fractures within 24 h in Germany [15]. However, studies have shown malnutrition in 73% of patients with hip fracture [16, 17]. The perioperative hemoglobin level also shows significant impact on patients’ recovery [18].

It has also been shown that regional anesthesia is associated with lower odd of mortality when comparing with general anesthesia [19]. By calculating 90-day mortality and complication rates, orthopaedic surgeons can compare these data with the situation in their departments and act accordingly. It is also important to know how complications and mortality vary with operation time. Mortality increases if the operation time exceeds 86 min. With this knowledge, the surgeon should actively avoid delays and prolonged operation times. The findings of this study should be confirmed with further investigations determining the optimal operation time for patients with femoral neck fractures undergoing HHA.

In addition, patient age and male sex both were identified as a factor in increased mortality. Surgeons should be aware of the increased risk when operating on elderly male patients performing HHA. Interestingly, the experience of the surgeon also has an impact on mortality. The literature is contradictory on this point [20–22]. While some studies find no difference in surgeon experience and consider HHA to be an appropriate training procedure [21], other studies clearly show that a high level of experience in HHA does indeed lead to a better outcome [20, 22]. One possible explanation for these conflicting results is that training of new operators may vary considerably from hospital to hospital and from country to country. The ASA score of the operated patient and the time to surgery did not appear to have any influence on mortality and complications. In this context, the recommendation from an analysis of 178 HHA patients is to aim for surgical treatment within the first 24 h, but not at the expense of adequate patient preparation or neglecting the patient’s individual risk factors [23]. An analysis of 106,187 patients with proximal femur fractures between 2015 and 2017 revealed interesting results [24]. Depending on the time of surgery, mortality was increased for pertochanteric fractures treated with osteosynthesis, but not for femoral neck fractures treated with osteosynthesis or HHA [24]. In contrast, the total number of complications was significantly increased by delaying surgery [24, 25]. For femoral neck fractures treated with HHA, the risk of complications increased by 121% and 142% if the time to surgery was 3 days respectively 4–7 days [24]. Based on these finding surgery should be performed by an experienced surgeon and the patients should be prepared for surgery as good as possible.

The results of the present study were discussed in the light of another important complication. BCIS is characterized by circulatory instability to the point of resuscitation that occurs during intraoperative cementing, prosthesis insertion or immediately postoperatively [26]. In our patient cohort, we analyzed the 50 deaths in more detail and found that five patients (10% of all deaths) had to be resuscitated intraoperatively or immediately postoperatively. These patients then died immediately or in the immediate aftermath. All five patients were in the group of patients who had received a cemented prosthesis, so BCIS is suspected as a possible cause of death. Orthopaedic experts are discussing a possible reduction in the risk of BCIS by recommending a moderate injection of bone cement into the medullary cavity. An equally moderate insertion of the prosthesis stem into the medullary cavity may help to reduce the risk. The aim is to avoid pressure peaks that could cause solid components from the medullary cavity to enter the bloodstream and trigger BCIS [26, 27]. A closer look at the operation times of these five patients showed no relevant deviations from the average operation time.

There are several limitations. The study was retrospectively designed using data on complications and mortality extracted from discharge letters, quality assurance data sheets, and patient administration software. A potential risk remains for coding errors. The quality assurance database only allowed a 90-days outcome analysis and thus mid-term or long-term complications are missing. There may also be other confounding factors that were not assessed in this study.

## Conclusion

This retrospective data analysis found that the risk of a patient experiencing a complication was 2.2% higher for every minute increase in operation time. Patients with an operation time above the cut-off of 86 min had a 111.8% higher risk of mortality than those with an operation time below the cut-off. Other influencing factors that operators should be aware of include patient age, male sex and operator experience.

### Electronic supplementary material

Below is the link to the electronic supplementary material.

Supplementary Material 1

## Data Availability

No datasets were generated or analysed during the current study.

## Abbreviations

ASA: American Society of Anesthesiologists
BCIS: Bone cement implantation syndrome
BMI: Body mass index
CI: Confidence interval
HHA: Hip hemiarthroplasty
MD: Mean difference
NSQIP: National Surgical Quality Improvement Program
OR: Odds ratio
THA: Total hip arthroplasty
