# Supplementary material for: The influence of operation time for hip hemiarthroplasty on complication rates and mortality in patients with femoral neck fracture: a retrospective data analysis
Source: J Orthop Surg Res. 2024 May 27;19:311. doi: 10.1186/s13018-024-04797-7 (PMC11129483; doi:10.1186/s13018-024-04797-7)
Supplement: Supplementary file 1 — Supplementary Material 1 [file 13018_2024_4797_MOESM1_ESM.docx]

| Complication | | | | | |
| --- | --- | --- | --- | --- | --- |
| Parameter | Exponential beta | Beta | Standard error | z-value | p-value |
| Intercept | 0.001 | -7.257 | 2.489 | -2.916 | 0.0035 ** |
| Patient age (years) | 1.009 | 0.009 | 0.022 | 0.397 | 0.6916 |
| Senior operator: yes | 1.139 | 0.130 | 0.443 | 0.293 | 0.7693 |
| Sex: male | 1.661 | 0.508 | 0.345 | 1.473 | 0.1406 |
| ASA score | 1.935 | 0.660 | 0.343 | 1.926 | 0.0541 |
| Operation within 24h: yes | 1.202 | 0.184 | 0.365 | 0.505 | 0.6136 |
| Operation time (min.) | 1.022 | 0.021 | 0.010 | 2.094 | 0.0363 * |

Multiple logistic regression model for the complications, investigating the following factors: patient age, senior operator, sex, ASA score, time to operation, operation time.

| Complication | | | | | |
| --- | --- | --- | --- | --- | --- |
| Parameter | Exponential Beta | Beta | Standard error | z-value | p-value |
| Intercept | 0.002 | -6.253 | 1.489 | -4.200 | < 0.0001 *** |
| Patient age group: >80 years | 0.810 | -0.211 | 0.365 | -0.579 | 0.5628 |
| Senior operator: yes | 1.088 | 0.085 | 0.444 | 0.191 | 0.8488 |
| Sex: male | 1.599 | 0.469 | 0.342 | 1.371 | 0.1703 |
| ASA score | 1.964 | 0.675 | 0.345 | 1.959 | 0.0501 |
| Operation within 24h: yes | 1.193 | 0.176 | 0.365 | 0.483 | 0.6289 |
| Operation time (min.) | 1.020 | 0.020 | 0.010 | 1.967 | 0.0492 * |

Multiple logistic regression model for the complications, investigating the following factors: patient age group, senior operator, sex, ASA score, time to operation, operation time.

| Complication | | | | | |
| --- | --- | --- | --- | --- | --- |
| Parameter | Exponential Beta | Beta | Standard error | z-value | p-value |
| Intercept | 0.001 | -7.419 | 2.464 | -3.011 | 0.0026 ** |
| Patient age (years) | 1.010 | 0.010 | 0.022 | 0.436 | 0.6625 |
| Senior operator: yes | 1.166 | 0.154 | 0.441 | 0.348 | 0.7278 |
| Sex: male | 1.649 | 0.500 | 0.345 | 1.449 | 0.1473 |
| ASA score | 1.944 | 0.665 | 0.343 | 1.938 | 0.0527 |
| Operation within 24h: yes | 1.193 | 0.176 | 0.365 | 0.483 | 0.629 |
| Operation time rounded (min.) | 1.024 | 0.024 | 0.010 | 2.355 | 0.0185 * |

Multiple logistic regression model for the complications, investigating the following factors: patient age, senior operator, sex, ASA score, time to operation, operation time rounded.

| Complication | | | | | |
| --- | --- | --- | --- | --- | --- |
| Parameter | Exponential Beta | Beta | Standard error | z-value | p-value |
| Intercept | 0.002 | -6.352 | 1.460 | -4.352 | < 0.0001 *** |
| Age group: >80 years | 0.819 | -0.200 | 0.366 | -0.546 | 0.5853 |
| Senior operator: yes | 1.114 | 0.108 | 0.442 | 0.245 | 0.8063 |
| Sex: male | 1.584 | 0.460 | 0.343 | 1.342 | 0.1795 |
| ASA score | 1.974 | 0.680 | 0.345 | 1.972 | 0.0486 * |
| Operation within 24h: yes | 1.185 | 0.169 | 0.365 | 0.464 | 0.6427 |
| Operation time rounded (min.) | 1.022 | 0.022 | 0.010 | 2.227 | 0.0259 * |

Multiple logistic regression model for the complications, investigating the following factors: patient age group, senior operator, sex, ASA score, time to operation, operation time rounded.

| Mortality | | | | | |
| --- | --- | --- | --- | --- | --- |
| Parameter | Exponential Beta | Beta | Standard error | z-value | p-value |
| Intercept | 0.000 | -10.785 | 2.570 | -4.197 | < 0.0001 *** |
| Patient age (years) | 1.088 | 0.084 | 0.025 | 3.386 | 0.0007 *** |
| Senior operator: yes | 0.406 | -0.901 | 0.359 | -2.512 | 0.0120 * |
| Sex: male | 2.687 | 0.988 | 0.316 | 3.132 | 0.0017 ** |
| ASA score | 1.684 | 0.521 | 0.320 | 1.632 | 0.1027 |
| Operation within 24h: yes | 0.805 | -0.217 | 0.319 | -0.680 | 0.4968 |
| Operation time (min.) | 1.002 | 0.002 | 0.010 | 0.155 | 0.8769 |

Multiple logistic regression model for the mortality, investigating the following factors: patient age, senior operator, sex, ASA score, time to operation, operation time.

| Mortality | | | | | |
| --- | --- | --- | --- | --- | --- |
| Parameter | Exponential Beta | Beta | Standard error | z-value | p-value |
| Intercept | 0.008 | -4.882 | 1.433 | -3.408 | 0.0007 *** |
| Patient age group: >80 years | 4.929 | 1.595 | 0.540 | 2.956 | 0.0031 ** |
| Senior operator: yes | 0.385 | -0.955 | 0.357 | -2.679 | 0.0074 ** |
| Sex: male | 2.493 | 0.913 | 0.311 | 2.940 | 0.0033 ** |
| ASA score | 1.821 | 0.599 | 0.319 | 1.881 | 0.06 |
| Operation within 24h: yes | 0.827 | -0.189 | 0.318 | -0.596 | 0.551 |
| Operation time (min.) | 0.998 | -0.002 | 0.010 | -0.148 | 0.8827 |

Multiple logistic regression model for the mortality, investigating the following factors: patient age group, senior operator, sex, ASA score, time to operation, operation time.

| Mortality | | | | | |
| --- | --- | --- | --- | --- | --- |
| Parameter | Exponential Beta | Beta | Standard error | z-value | p-value |
| Intercept | 0.000 | -10.247 | 2.534 | -4.043 | < 0.0001 *** |
| Patient age (years) | 1.085 | 0.082 | 0.025 | 3.312 | 0.0009 *** |
| Senior operator: yes | 0.382 | -0.962 | 0.360 | -2.669 | 0.0076 ** |
| Sex: male | 2.715 | 0.999 | 0.316 | 3.163 | 0.0016 ** |
| ASA score | 1.684 | 0.521 | 0.320 | 1.629 | 0.1034 |
| Operation within 24h: yes | 0.809 | -0.212 | 0.320 | -0.664 | 0.5066 |
| Operation time rounded (min.) | 0.997 | -0.003 | 0.011 | -0.320 | 0.7489 |

Multiple logistic regression model for the mortality, investigating the following factors: patient age, senior operator, sex, ASA score, time to operation, operation time rounded.

| Mortality | | | | | |
| --- | --- | --- | --- | --- | --- |
| Parameter | Exponential Beta | Beta | StandardError | z value | p.value |
| Intercept | 0.011 | -4.513 | 1.399 | -3.227 | 0.0013 ** |
| Patient age group: >80 years | 4.831 | 1.575 | 0.539 | 2.922 | 0.0035 ** |
| Senior operator: yes | 0.362 | -1.017 | 0.359 | -2.833 | 0.0046 ** |
| Sex: male | 2.522 | 0.925 | 0.311 | 2.976 | 0.0029 ** |
| ASA score | 1.818 | 0.598 | 0.319 | 1.872 | 0.0611 |
| Operation within 24h: yes | 0.832 | -0.184 | 0.318 | -0.577 | 0.564 |
| Operation time rounded (min.) | 0.994 | -0.007 | 0.011 | -0.614 | 0.539 |

Multiple logistic regression model for the mortality, investigating the following factors: patient age group, senior operator, sex, ASA score, time to operation, operation time rounded.
